# SARS Clinical Features, United States, 2003

**DOI:** 10.3201/eid1101.040585

**Published:** 2005-01

**Authors:** Padmini Srikantiah, Myrna D. Charles, Sarah Reagan, Thomas A. Clark, Mathias W.R. Pletz, Priti R. Patel, Robert M. Hoekstra, Jairam Lingappa, John A. Jernigan, Marc Fischer, for the CDC SARS Clinical Investigation Team

**Affiliations:** * Centers for Disease Control and Prevention, Atlanta, Georgia, USA

**Keywords:** Severe acute respiratory syndrome, SARS, SARS-associated coronavirus, clinical features, United States, case-control, dispatch

## Abstract

We compared the clinical features of 8 U.S. case-patients with laboratory-confirmed severe acute respiratory syndrome (SARS) to 65 controls who tested negative for SARS coronavirus (SARS-CoV) infection. Shortness of breath, vomiting, diarrhea, progressive bilateral infiltrates on chest radiograph, and need for supplemental oxygen were significantly associated with confirmed SARS-CoV infection.

The clinical course and outcomes of cases of severe acute respiratory syndrome (SARS) in Asia and Canada have been well described (*1*–*6*). Most of these studies defined cases based on clinical and epidemiologic criteria with or without laboratory evidence of SARS-associated coronavirus (SARS-CoV) infection. In the event of a subsequent outbreak, distinguishing clinical features associated with SARS-CoV infection may help inform decisions regarding patient evaluation and infection control practices while laboratory results are pending. We describe the clinical characteristics of patients in the United States with laboratory-confirmed SARS and compare them to persons who tested negative for SARS-CoV but had similar illnesses

## The Study

We defined a case-patient as a U.S. resident who met the clinical and epidemiologic criteria for suspected or probable SARS and had laboratory evidence of SARS-CoV infection (*7*). L*a*boratory evidence of SARS-CoV infection was defined as 1) isolation of SARS-CoV, 2) detection of SARS-CoV RNA by polymerase chain reaction (PCR), or 3) detection of antibodies against SARS-CoV by using enzyme-linked immunosorbant assay or indirect fluorescent-antibody assay (*8*,*9*).

After obtaining verbal consent, health officials used a standard questionnaire to interview by telephone patients with suspected or probable SARS and their healthcare providers. Data collected included clinical symptoms, past medical history, relevant exposures, physical examination, radiographic and laboratory findings, and clinical course and outcome.

Case-patients with laboratory-confirmed SARS were compared to a convenience sample of persons who met the clinical and epidemiologic criteria for suspected or probable SARS but subsequently tested negative for SARS-CoV infection. Controls had negative findings on all testing performed for SARS-CoV, including the absence of antibody against the virus in convalescent-phase serum samples obtained >21 days after onset of symptoms. Statistical analysis was performed with SAS software version 8.2 (SAS Institute, Cary, NC). Univariate odds ratios, 95% confidence intervals, and p values for association were calculated by using exact likelihood methods.

We identified 8 case-patients with laboratory-confirmed SARS-CoV infection in the United States. Dates of onset of symptoms were from February 22 to May 24, 2003. The median age of case-patients was 43 years (range 22–53 years); 4 were women. Two case-patients were pregnant (8 weeks’ and 19 weeks’ gestation) at the onset of their illness. No other major underlying medical conditions were noted.

Seven case-patients reported travel to an area with community transmission of SARS in the 10 days before illness onset, including Hong Kong (n = 4), Toronto (n = 2), and Singapore (n = 1). One case-patient returned to the United States 13 days before illness onset after traveling to Hong Kong with her spouse, who was also a laboratory-confirmed SARS patient. Three (38%) patients visited a healthcare facility during their travel in the 10 days before illness onset, and 4 patients stayed at a hotel associated with a well-defined SARS cluster (*7*).

Over the course of their illness, findings suggestive of a lower respiratory tract infection developed in all 8 patients with laboratory-confirmed SARS; these findings included dyspnea (n = 8), rales (n = 5), and hypoxia (n = 5) (Table 1). Symptoms indicative of an upper respiratory tract infection, including rhinorrhea and sore throat, were reported less often. The most common symptoms at illness onset included fever (n = 8), chills (n = 6), and headache (n = 5). Four (50%) patients reported at least 1 respiratory symptom at illness onset. In the remaining 4 patients, respiratory symptoms began 3–7 days after illness onset. The median duration of symptoms before a patient sought medical evaluation was 6 days (range 3–14 days). When patients were first evaluated, the median recorded temperature was 38.6°C (range 37.0°C–40.0°C); the median recorded oxygen saturation on room air was 95% (range 87%–100%).

**Table 1:** Signs and symptoms of patients with laboratory-confirmed SARS-CoV infection, United States, 2003 (N = 8)*

Signs and symptoms	Any time during illness, n (%)	At illness onset, n (%)
Temperature >38.0°C	8 (100)	8 (100)
Room air oxygen saturation <94%	5 (63)	NA
Chills/rigors	7 (88)	6 (75)
Headache	6 (75)	5 (63)
Rhinorrhea	2 (25)	0 (0)
Sore throat	1 (13)	0 (0)
Cough	8 (100)	2 (25)
Sputum production	4 (50)	0 (0)
Dyspnea	8 (100)	1 (13)
Rales	5 (63)	NA
Vomiting	5 (63)	0 (0)
Diarrhea	6 (75)	0 (0)

*SARS-CoV, severe acute respiratory syndrome–associated coronavirus; NA, not available.

Gastrointestinal symptoms were also prominent. Six patients reported diarrhea, and 5 reported vomiting during the course of their illness. When present, diarrhea occurred a median of 3 days after onset (range 2–3 days) and was noted before (n = 4), or within 48 hours (n = 2) of receiving antimicrobial therapy. Vomiting began a median of 5 days after onset (range 3–9 days).

All 8 case-patients had radiographic evidence of pulmonary infiltrates during the course of their illness (Table 2). Bilateral pulmonary infiltrates developed in 7 patients during the course of illness with both interstitial and alveolar involvement. Of these, 6 demonstrated worsening chest radiographic findings in week 2 of illness.

**Table 2:** Clinical, radiographic, and laboratory features of patients with laboratory-confirmed SARS-CoV infection, United States, 2003 (N = 8)*†

Finding	n (%)
Radiographic findings	
Abnormal chest radiograph	8 (100)
Bilateral infiltrates	7 (88)
Prolonged progression of infiltrates‡	6 (75)
Pleural effusions	3 (38)
Acute respiratory distress syndrome	1 (13)
Laboratory findings	
Hematocrit <36%	2 (25)
Leukocyte count <4,000 cells/mm^3^	2 (25)
Absolute lymphocyte count <1,500 cells/mm^3^	7 (88)
Platelets <150,000/mm^3^	2 (25)
Clinical course and outcomes	
Hospitalized	7 (88)
Admitted to intensive care unit	2 (25)
Received supplemental oxygen	6 (75)
Required mechanical ventilation	1 (13)
Died	0 (0)

*Features present at any time during course of illness. †SARS-CoV, severe acute respiratory syndrome–associated coronavirus. ‡Radiographic worsening of infiltrates >7 days after onset of symptoms.

The first abnormal chest radiograph was obtained a median of 7 days after onset of symptoms (range 1–14 days). Six patients had an abnormal chest radiograph when first evaluated, including 3 with bilateral infiltrates. Two patients had unremarkable initial chest radiographs on days 6 and 8 after onset, respectively, but were subsequently noted to have infiltrates on chest imaging obtained on days 8 and 11 of their illness.

During the course of their illness, all 8 case-patients received antibacterial therapy. Three patients also received oseltamivir; none was treated with ribavirin. One patient received corticosteroids. Seven patients were hospitalized for a median of 8 days (range 6–15 days). Two patients were admitted to the intensive care unit for 7 and 9 days, respectively; no deaths occurred (Table 2).

Antibodies against SARS-CoV developed in all 8 patients; 3 had positive PCR findings in clinical specimens (1 sputum and 2 stool specimens) (*7*). Variable levels of clinical laboratory testing were performed (Table 2).

The 8 patients with laboratory-confirmed SARS were compared to 65 SARS-CoV–negative controls (>18 years old), of whom 14 (22%) had radiographic evidence of pneumonia. Forty-four (68%) controls tested negative for antibodies to SARS-CoV on serum obtained >28 days after symptom onset; the remaining 21 (32%) controls had a negative serologic finding for SARS-CoV 22–28 days after illness onset.

Patients were similar to controls with regard to age and sex. Fifty-eight (89%) controls reported travel to an area with community transmission of SARS in the 10 days before illness onset. However, patients were significantly more likely than controls to have visited a healthcare facility during their travel (3/8 versus 4/65; p = 0.03) or to have stayed at the hotel associated with the SARS cluster (3/8 versus 1/65; p < 0.01).

Univariate analysis of clinical features showed that dyspnea, hypoxia, rales, vomiting, and diarrhea were more common among SARS-CoV–positive patients than SARS-CoV–negative controls (Table 3). Case-patients were also significantly more likely than controls to report fever as an initial symptom (8/8 versus 29/65; p < 0.01) and to have an abnormal chest radiograph at the time of first evaluation (6/8 versus 12/52; p < 0.01). When the analysis was limited to patients with radiographic evidence of pneumonia, dyspnea and vomiting remained associated with SARS-CoV infection. In addition, SARS-CoV–positive cases were significantly more likely to have bilateral multifocal infiltrates (7/8 cases versus 4/14 controls; p = 0.02) and radiographic progression of pulmonary infiltrates into week 2 of illness (6/8 cases versus 0/14 controls; p < 0.01).

**Table 3:** Univariate analysis for distinguishing clinical features of SARS-CoV–positive cases from SARS-CoV–negative controls, United States, 2003*

Clinical feature	SARS-CoV–positive cases (N = 8), n (%)	SARS-CoV–negative controls (N = 65), n (%)	OR (95% CI)	p value
Dyspnea	8 (100)	32 (49)	10.9 (1.6, ∞)	0.01
Hypoxia	5 (63)	9 (14)	9.9 (1.6, 75.0)	0.01
Rales	5 (63)	15 (23)	5.4 (0.9, 38.9)	0.06
Sore throat	1 (13)	39 (60)	0.1 (0.0, 0.8)	0.03
Vomiting	5 (63)	3 (5)	30.5 (4.0, 315.4)	< 0.01
Diarrhea	6 (75)	18 (28)	7.6 (1.2, 83.6)	0.02
Radiographic evidence of infiltrates at first evaluation	6 (75)	12† (23)	10.0 (1.8, 56.2)	< 0.01
Lymphopenia	7 (88)	24‡ (53)	6.0 (0.7, 288.9)	> 0.10

*SARS-CoV, severe acute respiratory syndrome–associated coronavirus; OR, odds ratio; CI, confidence interval. †Among 52 controls who had chest radiograph performed. ‡Among 45 controls who had complete blood counts performed.

## Conclusions

We compared the 8 U.S. patients with laboratory-confirmed SARS to SARS-CoV–negative controls who met the clinical and epidemiologic criteria for suspected or probable SARS. Our findings indicate that SARS-CoV infection is associated with significant lower respiratory tract disease. Patients with laboratory-confirmed SARS were more likely than controls to have dyspnea, hypoxia, and rales. Patients were also more likely than controls to have an abnormal chest radiograph at the time of first evaluation. These clinical findings are similar to those reported in case series from Asia and Canada, and contrast the clinical manifestations of SARS-CoV with most viral respiratory pathogens including other human coronaviruses (*1*–*5*,*10*). When compared to controls with radiographic evidence of pneumonia, patients with SARS were more likely to manifest dyspnea and progressive bilateral pulmonary infiltrates. This radiographic progression to multifocal infiltrates has been a prominent finding in several previous studies and may prove to be a hallmark feature of the later stages of this disease (*1*–*3*,*6*,*11*). Among U.S. case-patients, diarrhea and vomiting were also significantly associated with SARS-CoV infection. While gastrointestinal symptoms were a relatively uncommon feature in some previous reports (*1*,*3*), diarrhea was frequently reported in other case series, including a major community outbreak at a Hong Kong apartment block (*2*,*4*,*5*,*12*).

Although previous studies have described the clinical features of patients with laboratory-confirmed SARS, none compared the characteristics of these patients with SARS-CoV–negative controls. Our findings suggest that the combination of gastrointestinal symptoms, dyspnea, and bilateral pulmonary infiltrates may warrant a higher level of suspicion for SARS-CoV infection. By contrast, patients with findings of only upper respiratory tract infection may be unlikely to have SARS. Although moderate lymphopenia was prominent among U.S. case-patients, it was also a fairly common finding among controls who likely had other viral sources of infection. The small number of persons with laboratory-confirmed SARS in the United States limited our power to identify independent clinical predictors of SARS-CoV infection. Further data are needed to describe the full clinical spectrum of SARS-CoV infection and to clarify when specific clinical findings are most likely to occur during the course of illness (*13*,*14*).

Early recognition of possible SARS-CoV infection and rapid initiation of infection control precautions are currently the most important strategies for controlling SARS (*15*). Identifying persons who warrant further investigation for SARS-CoV infection may be difficult on the basis of clinical symptoms alone, especially early in the course of illness. Appropriate preparedness for SARS will thus require vigilant clinicians and public health officials to integrate timely epidemiologic information, astute clinical evaluation, and improved laboratory diagnostic tools.
